# Effect of Fabric Architecture on Tensile Behaviour of the High-Molecular-Weight Polyethylene 3-Dimensional Interlock Composite Reinforcements

**DOI:** 10.3390/polym12051045

**Published:** 2020-05-02

**Authors:** Mengru Li, Peng Wang, François Boussu, Damien Soulat

**Affiliations:** 1 University of Lille, Ensait, Gemtex, F-59000 Roubaix, France; mengru.li@ensait.fr (M.L.); francois.boussu@ensait.fr (F.B.); damien.soulat@ensait.fr (D.S.); 2 University of Haute-Alsace, Ensisa, Lpmt, F-68000 Mulhouse, France

**Keywords:** fabric/textiles, HMWPE, 3D warp interlock fabrics, mechanical properties

## Abstract

As promising fibrous reinforcements in the thick composites manufacturing, 3-dimensional warp interlock fabrics (3DWIFs) are recognised more and more in the industry for their outstanding mechanical properties compared to the 2D laminates. The present work shows the influence of the fabric’s architecture on the tensile behaviour of 3DWIFs. Five kinds of 3D fabrics with different interlock structures have been designed according to the main category of binding warp yarn evolution. These five 3DWIFs, containing both binding and stuffer warp yarns and produced with the same warp and weft densities, are experimentally tested via uniaxial tensile tests. The experimental results of the different 3DWIFs have been compared to find the optimal solution based on several mechanical performances. Fabric structures have an impact on tensile properties both in the warp and weft directions. Furthermore, other influential factors, for example, the yarn crimps during the weaving process and the crimp angles of binding warp yarns in 3DWIFs, are investigated and discussed in the paper. The influence of the total crimp angles related to the binding path on the tensile properties of 3DWIFs via the inter yarns friction is summarised.

## 1. Introduction

3-Dimensional warp interlock fabrics (3DWIFs) have gained tremendous attention for use as reinforcement for composites over the past decades [1,2]. As a new class of lightweight materials, 3DWIFs have been increasingly used for composite reinforcements with wide applications in military, aircraft, automobile, aerospace and medical devices [3,4,5,6,7,8,9,10]. This illustrates that the use of 3DWIFs as fibrous reinforcement of composites possesses outstanding mechanical properties and other advantages compared to 2D laminates. For example, Sun et al. [11] showed that there is no delamination between each layer of the 3D orthogonal woven composites under high strain rate compressive loading because of the binds of Z-yarns comparing with 2D plain woven basalt composites. As another example, Ivanov et al. [12] demonstrated that the non-crimp 3D orthogonal woven composites have significantly higher in-plane strengths, failure strains and damage initiation thresholds than their 2D woven laminated counterpart. 

A variety of researchers have mainly attached importance to the mechanical properties of the 3D warp interlock composites [13,14]. However, the potential of 3DWIFs preforms has not been completely explored and has not been widely conducted yet in the open literature. In other words, the mechanical behaviours of dry 3DWIFs as reinforcements are yet unclear. Due to this lack of knowledge and experience on these new materials, it could be difficult to predict their final behaviour and express their material properties. This can be attributed to the complexity of the different parameter combinations of the 3DWIFs. Besides, tensile behaviour analysis has become increasingly significant in the understanding of the material mechanical properties. Huang et al. [15] reported that the tensile properties of a woven structure were mainly determined by the waviness of the fibre in the loading direction. Hou et al. [7] reported the tensile behaviour of the 3D angle-interlock woven (3D-A) fabric under high strain rates which had not been reported, either in experimental testing or in theoretical modelling. Besides, the 3D orthogonal structure with the least crimp in the loading direction exhibited the highest tensile strength [15]. Bandaru et al. [1] reported that all the 3D fabrics exhibited typical tensile response in the warp direction, which was similar to results mentioned in Boussu et al. [16]. It was also demonstrated that the strength and failure strain in the weft direction were greater than in the warp direction. 

Thus, an investigative study to understand the effect of structure on the tensile behaviour of 3DWIFs is needed to help the designers to create new structures using 3DWIFs as fibrous reinforcement for composite material. Many parameters are involved in determining the tensile properties of the 3DWIFs. From the review of the previous studies, the effects on tensile mechanical properties were studied from the following aspects; the materials used [1,17], the structures [18], the weaving parameters [16,19,20,21], etc. However, the effect of architecture on the tensile properties of dry 3DWIFs is still not clearly known. In the present study, different 3DWIFs architectures are designed following the four main categories of 3DWIFs: Angle/Through-the-thickness binding (A/T), Angle/Layer-to-layer binding (A/L), Orthogonal/Through-the-thickness binding (O/T) and Orthogonal/Layer-to-layer binding (O/L). Five 3DWIFs with the same warp and weft densities were manufactured. The tensile properties and physical measurements are performed to understand their mechanical behaviour with respect to their architecture linked to the process and product parameters. The influences of the crimp angles and the binding path in the 3D warp interlock structure are also discussed. 

## 2. Materials and Methods 

### 2.1. Tested Three-Dimensional Warp Interlock Fabrics

**Figure 1:**
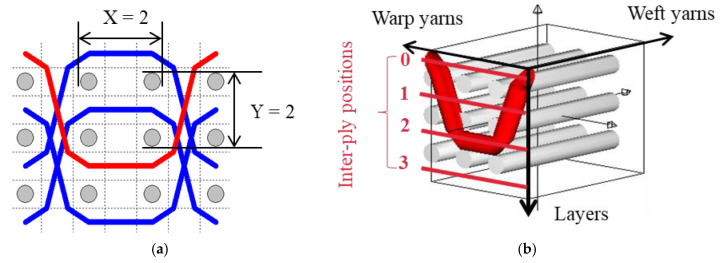
Representation sample of 3DWIF architectures based on the binding step and depth and the number of layers, warp binding path (X = 2) and binding depth (Y = 2): (**a**) cross-sectional weft yarns view; (**b**) 3D view.

The five different structures 3DWIFs were manufactured with high-molecular-weight polyethylene (HMWPE) yarns (Honeywell, Charlotte, North Carolina, USA) and with the same warp (10 ends/cm) and weft densities (40 picks/cm). The linear density is tested and characterized by the skein method based on ASTM D 1907/D 1907M. The density is obtained from the manufacturer. The tensile properties of HMWPE yarns with 50 twists per meter were performed by MTS Criterion Testing Systems. The main physical properties of HMWPE yarn are noted in Table 1. The definition of the structure is shown in Table 2. All cross-section weft yarns views and 3D views of 3DWIFs are designed by Wisetex software. In 3DWIF, the binding step (X) represents the number of weft yarns between two interlacing points of the binding warp yarn located in the same layer, and the binding depth (Y) represents the number of weft yarns layers linked with the binding warp yarn. Figure 1 shows a 3DWIF example where both the value of the binding step (X) and the binding depth (Y) are equal to 2. The weft yarns determine the number of layers of the 3D warp interlock structure and mostly provide the transverse mechanical properties of multi-layer fabric [22]. It can be seen that all the 3DWIFs samples have the same number of layers, warp and weft linear densities of tows and the same ratio of binding and stuffer warp yarns inside the structure. Therefore, all the basic structural parameters were kept the same for all the fabrics except for the binding step (X) and binding depth (Y). As for the F1 and F2 fabrics, the minimum binding depth was chosen (Y = 2) to design A/L and O/L 3D warp interlock architecture, and for F3 fabric, the binding depth was increased (Y = 3). The A/T and O/T interlock weave, which are the structures of F4 and F5 fabrics, have the maximum binding depth of binding warp yarns (Y = 4). Regarding the binding step, F1 and F2 fabrics have the same binding step (X = 3), and F3, F4 and F5 fabrics have the same binding step (X = 5). 

The main process parameters are given in Table 3. All the 3DWIFs were manufactured on the same dobby loom (Lindauer DORNIER GmbH, Rickenbach, Lindau, Germany). For all the fabrics, the average thickness of the specimens was precisely determined using an electromagnetic sensor thickness measuring apparatus based on the standard NF EN ISO 5084, and the areal densities were measured according to NF EN 12127. Thicknesses of all the fabrics decrease from F1 to F5 fabrics. This implies that the F1 fabric has the least compact structure and F5 fabric the most compact one. 

In the system of the 3DWIFs, stuffer warp yarns are selected by the heddles of the weaving loom and contribute to the longitudinal mechanical properties of the multi-layer fabric, and the binding warp yarns allow linking the various fabric layers in the thickness direction. The location of binding warp yarn plays a role in determining the thickness of the reinforcements. It helps to maintain cohesion throughout the woven structure according to their density inside the multi-layer structure, and thus contributes to increasing significantly the interlaminar resistance [22]. There is no doubt that tow waviness also influences the thickness of the dry fabric (through undulation frequency and amplitude) as described in [23]. Moreover, reinforcement in the thickness directly influences the fibre volume fraction of 3DWIFs, as all the reinforcements have similar warp and weft tow linear densities as well as equal warp density (ends/cm) and weft densities (picks/cm). Therefore, no matter the warp and weft densities, the fibre volume fractions increase from the F1 fabric to F5 fabric, mainly due to the 3D woven architecture, which allows better compaction for F5 fabric compared to other fabrics. In addition, there is only a slight difference between the five of them regarding the areal densities due to their different crimp values. The F5 fabric has the least thickness and the greatest fibre volume fraction, whereas the F1 fabric has the greatest thickness and the least value of fibre volume fraction.

Fibre volume fractions (FVF) are calculated by using the equation given below,

(1) $$FVF=\frac{W_{fabric}}{1000\times \rho \times T}\times 100\%$$

where *W*_fabric_ is the areal weight of the fabric reinforcement, *ρ* is the HMWPE fibre density (0.97 g/cm^3^) and *T* is the thickness of the fabric. 

### 2.2. Tensile Property Characterisation 

**Figure 2:**
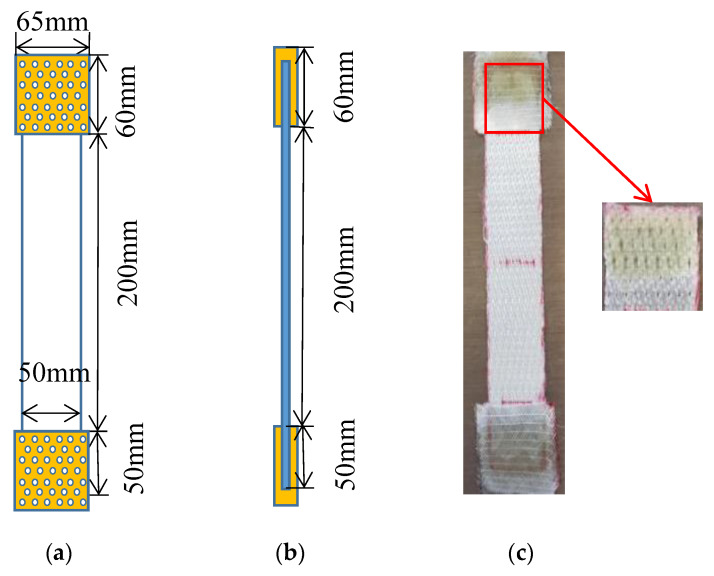
Methods of avoiding testing slippage by adhesive bonding on both ends with an extension of 50 mm: (**a**) the front photographic and schematic view; (**b**) schematic view of side plan; (**c**) actual operation.

The tensile tests in the present study are divided into two types: for a single HMWPE yarn and the 3D warp interlock fabric. The tensile properties of the different twisted HMWPE yarns were tested according to the ISO 2062 (1993) standard. The rectangular samples with the surface dimensions of 300 × 50 mm^2^ are submitted to tensile characterisation at the dry fabric scale. To avoid the slippage between the fabric sample and the tensile bench clamping jaws, adhesive bonding on both ends with an extension of 50 mm has been added by using epoxy resin to strengthen the part to be clamped. As the non-polar and chemical inertness of ultrahigh molecular weight polyethylene (UHMWPE) fibre surfaces inherently results in poor adhesion properties with most polymers [24,25,26,27], some little holes were made by sharp needles in both clamping fabric zone during the preparation process (Figure 2) to increase the adhesion of the epoxy resin on the UHMWPE fibres surfaces and finally to ensure zero slippage observed in the test. Tensile tests of the 3D warp interlock fabrics were performed according to the EN ISO 13 934-1 standard. Five tests for each architecture were performed in both warp and weft directions. The nominal length between the two clamps of the tensile bench was 200 mm at a speed of 100 mm/min at room temperature conditions (20 °C and 65% HR). 

### 2.3. Yarn Crimp Test

To analyse the influence of the yarn’s crimp on the tensile behaviour of the 3DWIFs, the yarn crimp tests according to ASTM D 3883 are proposed with yarn samples 10 cm in length. These tests are carried out for ten yarns of each 3DWIF structure carefully extracted from the dry 3DWIF samples.

## 3. Results and Discussion

### 3.1. Tensile Behaviour of a Single Yarn

**Figure 3:**
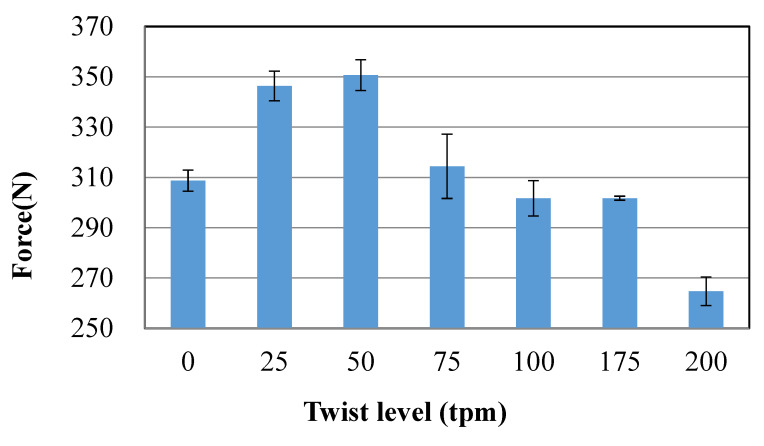
The ultimate forces of different twists HMWPE yarns.

Figure 3 shows the breaking tensile load of different twists HMWPE yarns. It can be observed that at the weak twist level (0–50 twist/m), the breaking tensile load increases with the increasing of yarn twist. This phenomenon agrees well with the work reported in [28], which illustrated that the strength of high-performance fibre yarns can be improved by a slight twist. The increase in strength is mainly due to an interlocking mechanism where the filaments are held together by radial forces and frictions, which, in effect, enables a single fibre to fail more than once. However, the greater the twist value is, the less the maximum tensile load is. This means there is a decrease in the breaking strength of the HMWPE yarns after exceeding a certain value of twist (the critical twist, 50 tpm in the present case). As the yarn twist value increases, the inclination of the fibre to the axis of the yarn increases, while the strength of the fibre can withstand the axial force of the yarn. Moreover, if the twist of yarn is too large, the fibre stress distribution inside and outside the yarn will be uneven, which will aggravate the different times of fibre breakage. Therefore, the parameters of 50 twists/m and “Z” twist were applied on the HMWPE yarns for the manufacturing of the 3D warp interlock fabrics.

**Figure 4:**
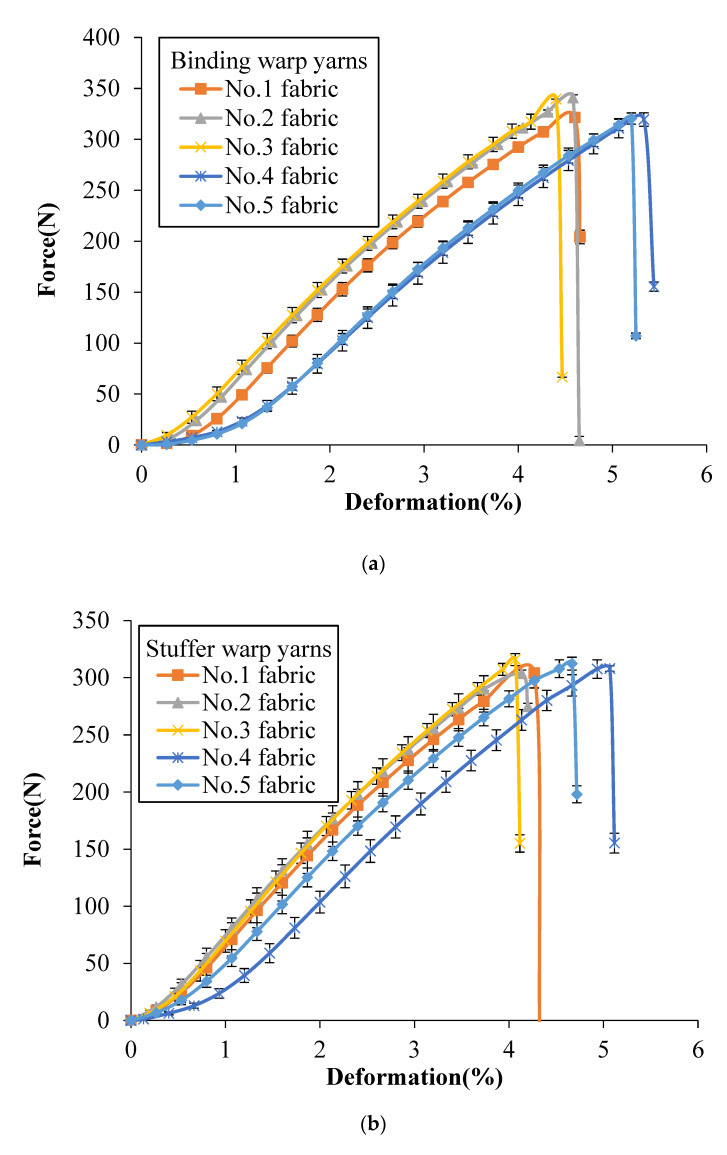

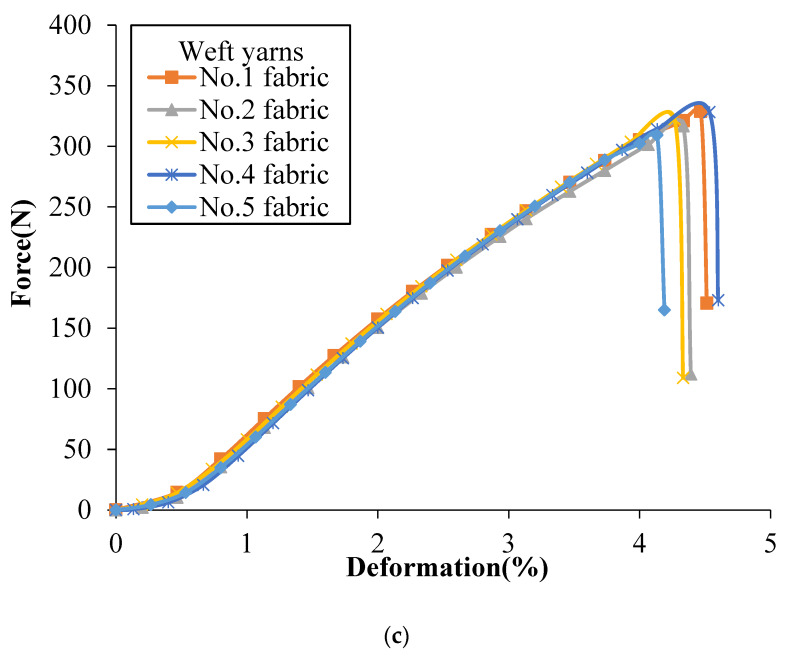
Tensile force vs. deformation curves of a single yarn after weaving in different 3DWIFs: (**a**) binding warp, (**b**) stuffer warp and (**c**) weft yarns.

Figure 4 shows the tensile force vs. deformation curves of the single yarns after weaving obtained from binding warp yarns, stuffer warp yarns and weft yarns, respectively, of different groups that each group of yarns providing a specific function within the structure. Similar nonlinear progression can be observed as the yarns are folded in the shape of perpendicular yarns, which is obvious in the 3D woven fabric yarns. It can be seen that no matter which architectures the yarns come from, the binding warp yarns have the highest breaking strength, followed by weft yarns and the stuffer warp yarns have the smallest breaking strength, which can be confirmed by comparing binding warp yarns, stuffer warp yarns and weft yarns from each fabric, respectively. Besides, on the whole, the binding warp yarns system has the highest breaking strength, the breaking strength of weft yarns system comes second and the stuffer warp yarns have the lowest breaking strength, which can be confirmed by calculating the average peak value (330 ± 10 N, 309 ± 6 N and 319 ± 9 N) of binding warp yarns, stuffer warp yarns and weft yarns from different fabrics, respectively. Note that the breaking load of a single yarn after weaving for different tested fabrics is reduced compared to the yarn before weaving (350.69 ± 6.13 N shown in Table 1). This is probably due to the friction during the weaving process that the binding warp yarns appear less affected by the weaving process than the stuffer warp yarns.

Slippage and breakage of fibres are some of the major causes of failure in yarns. As mentioned before, the HMWPE yarns have been twisted to increase their strengths. Filaments outside of the warp yarns are subjected to contact frictions which are between the yarns and with the heddles of dobby frame and the weaving reed during the weaving process. The curves in Figure 4c are completely coincident as all the fabrics are woven with twill surface pattern. Similarly, the weft yarns have no contact frictions with the heddles and weaving reed during their insertions. This is the reason why the weft yarns have larger ultimate forces than yarns from binding warp yarns and stuffer warp yarns. 

### 3.2. Yarn Crimp Properties

Table 4 shows the average crimp values of warp and weft yarns located in different layers for different dry 3DWIFs. The binding and stuffer warp yarns will not have the same yarn crimp value due to their different types of length consumption. It can be noticed that both warp and weft yarns crimp values of F4 fabric are the highest compared to others. However, the yarn crimps of F5 fabric have a great difference between binding (4.4 ± 1.06%) and stuffer warp yarns (0.95 ± 0.44%); crimps of the binding warp yarns are almost 5 times larger than the ones of stuffer warp yarns. The possible reason to explain the main difference in behaviour of the F5 structure (A-T fabric) compared to the other fabrics can be found in the highest difference of the crimp values of the binding and stuffer warp yarns, respectively. Then, during the tensile test, the two types of yarns respond to the load at the same time, and thus do not compensate for their weakness, revealing that the architecture parameters defining the evolution of warp yarns inside the 3D warp interlock fabric play an important role in the final mechanical behaviour of the 3D woven structure. 

**Figure 5:**
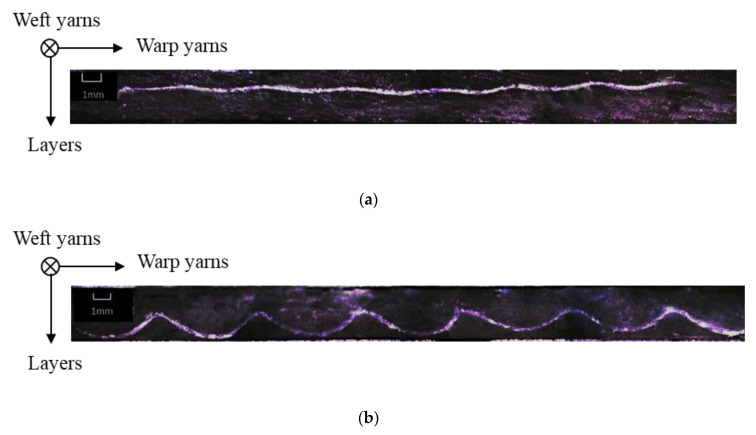
Micro-observation of the cross section of a single binding warp yarn in F2 (**a**) and F4 (**b**) fabrics.

Following the increase in binding depth from F1 to F5, the yarn crimps of both binding and stuffer warp yarns generally increase. Weft yarn crimp is almost the same for F1 and F3, and F4 and F5, respectively. The F2 fabric shows a slightly lower degree of undulation in weft tows. This lower crimp in the weft direction for the F2 fabrics does not affect the decreasing trend of total areal weight that decreases with the binding depth of warp tows, from F1 to F5 fabrics. The micro-observation of the weft cross-section can highlight the binding warp yarn evolution inside the fabric structure (shown in Table 2). Figure 5 shows the comparison of the single binding yarn cross-sectional shapes of F2 and F4 fabrics. There is a significant difference in the morphology between two binding warp yarns from different fabrics. The binding warp yarns in F4 fabric have important curvature and the crimp angle is much larger than the one of F2 fabric. By contrast, the binding warp yarns in F2 are almost straight in the fabric, which is in accordance with the results noted in Table 4. 

### 3.3. Tensile Behaviour of 3DWIFs 

**Figure 6:**
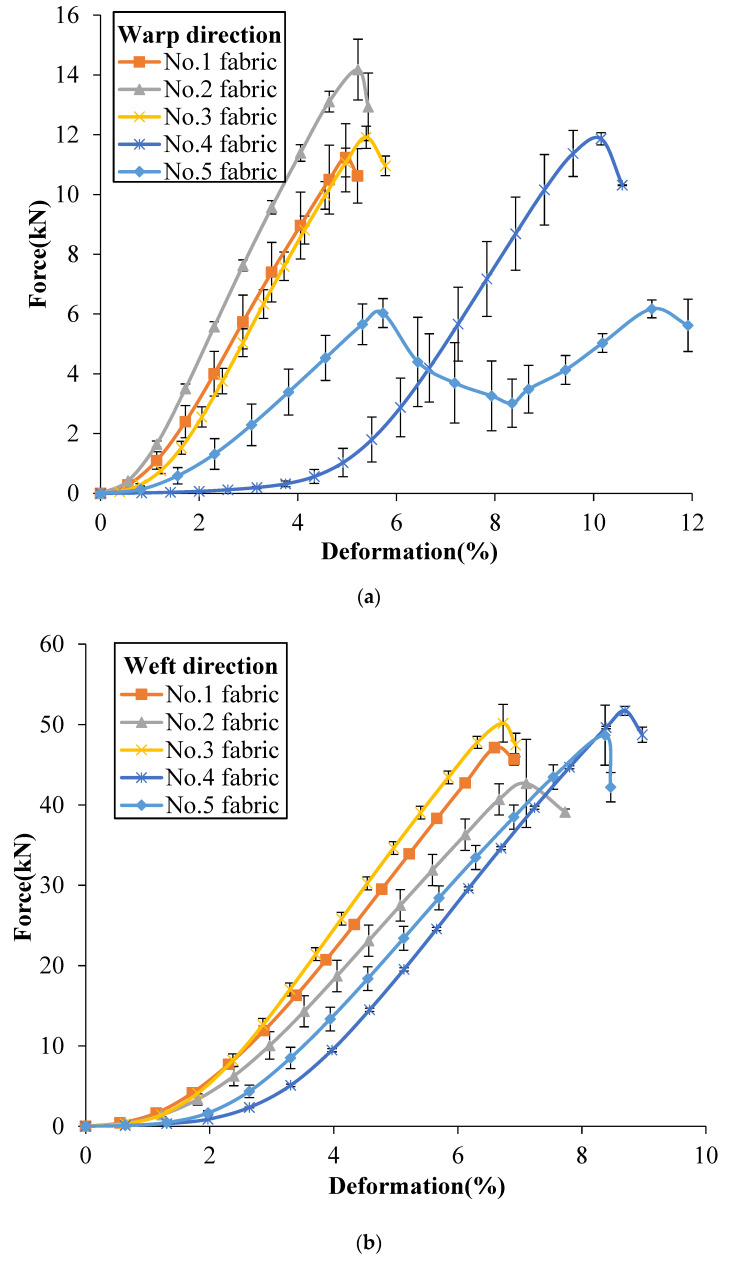
Tensile force/tensile deformation curves of 3DWIFs in warp (**a**) and weft (**b**) directions.

The tensile properties of the tested 3DWIFs were measured as a function of both strain and damage accumulation. Figure 6a,b show a comparison of the typical force–deformation response of five 3DWIFs in the warp and weft directions, respectively. As for the tensile behaviour, a nonlinear progression can be observed in both the warp and weft directions. The tensile curves can be generally divided into two parts: The first part is concerned with the alignment of yarns in the fabric structure when the deformation starts to increase slowly with the small amount of increasing tensile loads. It can be remarked a very important yarns alignment in the tensile process of F4 fabric due to the largest yarn crimps average values (Table 4). The extension of yarns can be observed in the second part; the curves of the F1, F2, F3 and F5 fabrics reveal linear progression where real straightening of yarn occurred with the rapid increase of tensile loads. 

In Figure 6a, the slope of the initial linear region of the F1, F3 and F4 fabrics was very similar. The extension slope is slightly smaller than the one of the F2 fabric but more important than the two slopes of the F5 fabric. The F5 fabric exhibited two peaks in the extension stage in the warp direction, as the average yarn crimp values of binding warp yarns were several times larger than the yarn crimp values of stuffer yarns. As presented in the literature [1,16], the three-dimensional angle interlock (3D-A) fabrics have the same typical tensile response (two peaks) in the warp direction. In the F5 fabric structure, the number of binding warp yarns is the same as one of the stuffer warp yarns (25 ends). It leads to an identical peak and the same tensile slope in two extension stages. The first load drop may occur exactly and the other part of the yarns moved relatively freely after one part of the warp yarns failed. The first peak indicates the strength of the stuffer yarns, and the second peak indicates the strength of the binding warp yarns finally aligned after elongation of the 3D fabric structure. However, there were no clear and distinguish peaks in other fabrics as the yarn crimps were similar between the binding and stuffer warp yarns in these fabrics, as shown in Table 5. 

Compared to the tensile results in the warp direction, the tensile curves in the weft direction of the different 3D warp interlock fabrics are quite similar. Following a small yarn alignment stage, the linear yarns extension stage reveals a quasi-identical slope. Bandaru et al. [1] reported a similar type of tensile response in the weft direction of 3D angle fabrics. The tensile resistance was generally greater in the weft direction as compared to warp direction in the case of all fabrics due to the main difference between end and pick densities [17]. The weft density was ~4 times larger than warp density. Another reason is that during weft insertion through a shuttle, weft tows do not undergo similar deterioration as warp tows during the weaving process [23].

As presented in Table 5, the failure strain was different in both warp and weft directions. It could be noticed that the value of elongation in the weft direction is higher than the corresponding in the warp direction for all the tested fabrics, except for the F4 fabric, which is due to inter-yarn friction provided by more HMWPE yarns in the weft direction. The F4 and F5 fabrics had higher failure strain in the warp direction, indicating that higher failure strain can be achieved by the A/T or O/T types of 3DWIFs structures, which means that through-the-thickness structures contribute to the higher breaking strain.

From a comparison of tensile response among these 3DWIFs (Figure 6), it was clear that the fabric architecture played an important role in the tensile behaviour of fabrics. Hereafter, Table 5 shows the influence of the fabric architecture on the tensile properties including tensile strength and failure strain of different fabrics. The F2 fabric shows the highest maximum tensile load in the warp direction and the lowest maximum tensile load in the weft direction. F4 fabric presents a weaker maximum tensile load at the highest strain than the other tested fabrics. This stage is then described as a stick-slip type or yarn translation [29]. 

### 3.4. Inter-Yarn Frictions during the Tensile Test of 3DWIFs

**Figure 7:**
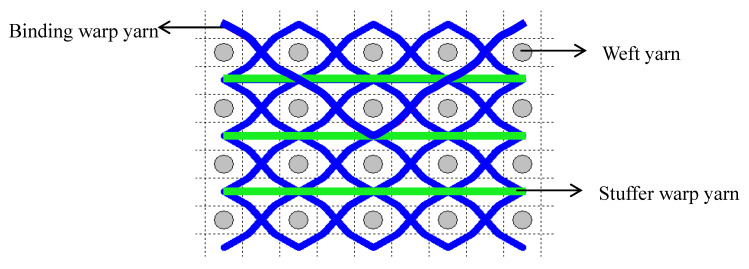
Example of the contact surface between the stuffer and weft yarns (in F1 fabric structure).

Compared to the weft direction, the tensile behaviour in the warp direction for 3DWIFs is more complex and interesting. During the tensile test in the warp direction, the work made through the clamping force can be described by Equation (2). $W_{m}$ is the work performed by the tensile machine, and $\;W_{e}^{B}$ and $W_{e}^{S}$ represent the work concerning the extension of the binding and stuffer warp yarns, respectively. $W_{f}^{B}$ and $W_{f}^{S}$ are the work related to the friction effect of the binding and stuffer warp yarns, respectively, in the yarns alignment stage (the inter-yarn sliding). Theoretically, the contact surface between the stuffer and weft yarns is very small (Figure 7) [1,30]. Consequently, $W_{f}^{S}$ can be neglected in Equation (2) and the details are developed in Equation (3). Therefore, the friction load of binding warp yarns ${\bar{F}}_{f}$ can be finally described by Equations (4) and (5),

(2) $$W_{m}=W_{e}^{B}+W_{e}^{S}+W_{f}^{B}+W_{f}^{S}$$

(3) $$\int_{0}^{u}F_{m}du=\sum_{1}^{n_{B}}\int_{u_{B}}^{u}F_{e}^{B}du+\sum_{1}^{n_{S}}\int_{u_{S}}^{u}F_{e}^{S}du+\sum_{1}^{n_{B}}{\bar{f}}_{f}\cdot u_{B}$$

(4) $${\bar{f}}_{f}=\frac{\int_{0}^{u}F_{m}du-\sum_{1}^{n_{B}}\int_{u_{B}}^{u}F_{e}^{B}du-\sum_{1}^{n_{S}}\int_{u_{S}}^{u}F_{e}^{S}du}{n_{B}\cdot u_{B}}$$

(5) $${\bar{F}}_{f}=n_{B}\cdot {\bar{f}}_{f}$$

where u (m) is the global displacement of the fabric (the displacement of the tensile machine clamps); $u_{B}$ (m) and $u_{S}$ (m) are the sliding of the binding and stuffer warp yarns, respectively, in the yarns alignment stage; $F_{e}^{B}$ (N) is the extension load of a single binding warp yarn shown in Figure 4; $F_{e}^{S}$ (N) is the extension load of a single stuffer warp yarn (Figure 4); $\bar{f_{f}}\;$ (N) and $\bar{F_{f}}$ (N) are the average friction loads during the tensile test on a single binding warp yarn and all the binding warp yarns, respectively; and $n_{1}$ and $n_{2}$ present the number of binding and stuffer warp yarns, respectively. 

**Figure 8:**
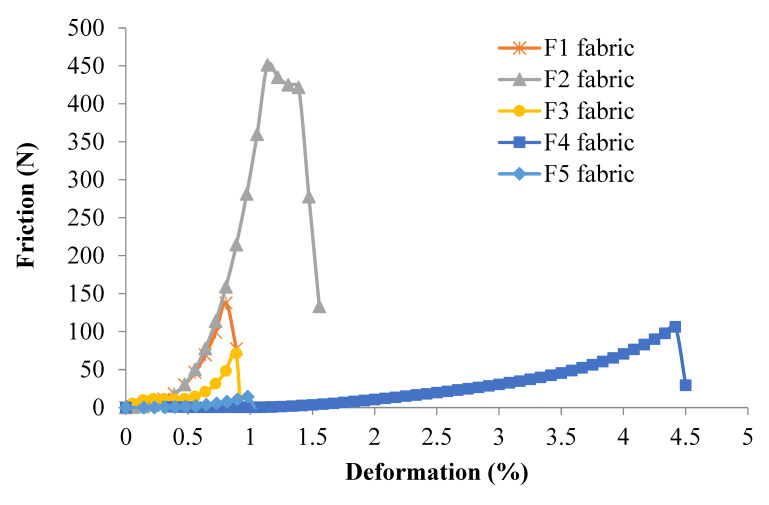
Average friction load–deformation of the binding warp yarns during the tensile tests of five different 3DWIFs samples.

Figure 8 shows the average friction force (${\bar{F}}_{f})$ on the binding warp yarns vs. the fabric deformation curves for all 3DWIFs samples during the tensile test. The binding warp yarns friction load ${\bar{F}}_{f}$ was calculated by Equation (5). Note that the F2 fabric has the highest friction load during the tensile test, which has a good agreement with the tensile results of the fabrics shown in Figure 6a. All the work done by the friction force happened before 1.6% of deformation, except the F4 fabric. The F4 fabric has the largest deformation (4.5%), which probably related to the largest yarn crimps in the 3D O-T structure. Therefore, it is clear that the work is done by binding warp yarns friction conducted in the crimp zone, which is displayed at the beginning of the 3DWIFs tensile tests. The trend of average friction load–deformation curves of the binding warp yarns during the tensile tests is similar to those shown in Figure 6a. The curve increased gradually at the beginning of the tensile test, the binding warp yarns only contact with the weft yarns without compression. Next, the weft yarns start to be compressed by binding warp yarns until it reaches the maximum. The binding warp yarns continued to be stretched until the strength reached its maximum value and the fabrics were broken. 

### 3.5. Geometrical Model and the Crimp Angle

**Figure 9:**
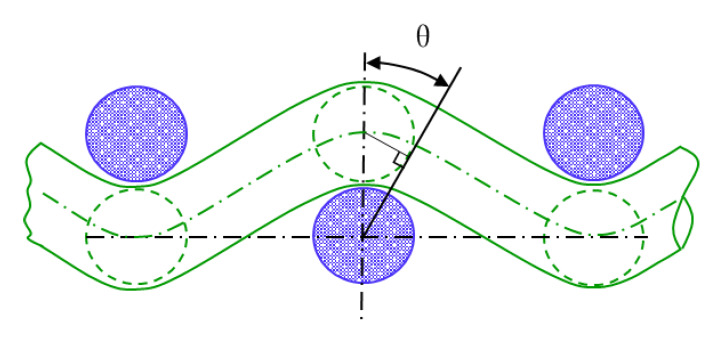
Definition of yarn crimp angle (θ) by using Peirce’s geometrical model of plain woven fabric.

Yarn interaction at the crossing points is the essential feature of woven fabric and affects more or less all the fabric properties [31]. The weave style and yarn interlacing strongly influence the tensile failure initiation of 2D fabrics [32]. The yarn interlacing method in which warp yarns cross under and over the weft yarns in 3DWIF is more complicated than in 2D fabrics. The arrangement of the yarn in the fabric can be mathematically described by Peirce’s geometrical model [33,34,35]. The definition of yarn crimp angle (θ) for the circular cross-sections and the flexible yarns is shown in Figure 9 using Peirce’s geometrical model. 

**Figure 10:**
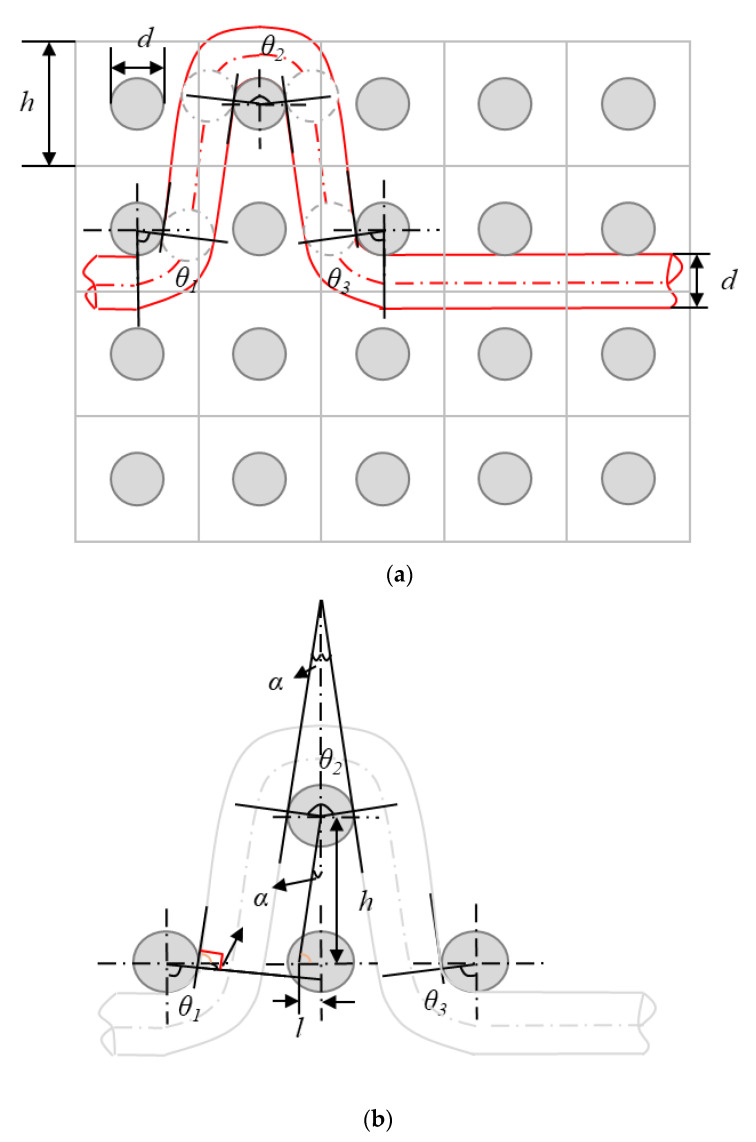
Schematic diagram (**a**) and geometrical model (**b**) of crimps angles (θ) and linking points among the warp and weft yarns in the unit cell of the F2 fabric.

In the present study, the yarn interlacing and crimp angle in 3DWIFs are described based on Peirce’s geometrical model for discussing and comparing the binding warp yarn frictions during the tensile test. Figure 10 gives an example of a schematic diagram and geometrical model of crimps angles (*θ_i_*) and linking points among the warp and weft yarns in the F2 fabric. It can be seen from the figure that this shape of the contact area between binding warp and weft yarns is the symmetrical structure. Assuming that the yarn is a cylinder, the yarn diameter (*d*) in mm can be calculated in the equation below,

(6) $$d=\sqrt{\frac{4T_{t}}{\pi \delta 10^{3}}}$$

where the yarn density is δ (g/cm^3^) and T_t_ is Tex of the yarn.

In Figure 10a, *h* presents the height of a cell, which is ~1 mm as the weft density is 42 picks/cm and there are ~16 picks weft yarns per unit cell in 4 layers (as shown in Figure 10a). Therefore, there are some gaps or spaces between the binding warp and weft yarns. As shown in Equation (7), *α* and *θ_1_* are the mutual complementary angles, and 2*α* and *θ_2_* are the supplementary angles. In Figure 10b, the crimp angle *θ_2_* is twice as large as *θ_1_* and *θ_3_*, where *θ_1_* and *θ_3_* are equal to each other. Finally, the crimp angles *θ* can be calculated by Equation (8), where *l* is approximately *d/2*.

(7) $$\{\begin{array}{c} \frac{\pi }{2}=\alpha +\theta_{1}\\ \frac{\pi }{2}=\alpha +\theta_{3}\;\\ \pi =2\alpha +\theta_{2}\\ \theta =\theta_{1}+\theta_{2}+\theta_{3}\\ \alpha =arctan\frac{l}{h} \end{array}$$

(8) $$\theta =2\pi -4\arctan(\frac{d}{2h})$$

According to the binding warp yarns interlacing in the fabrics and the geometrical model above, the crimp angles of the binding warp yarns and the linking points between the binding warp and weft yarns in different 3DWIF samples are calculated and shown in Table 6. The crimp angle depends mostly on the yarn interlacing and influences directly to the contact surface between binding warp and weft yarns. Table 6 shows the crimp angles and yarn interlacing shape of binding warp yarns in different fabrics per weave repeat. The warp yarn density (10.09 ± 0.13 ends/cm) and weft yarn density (42 ± 1.51 picks/cm) are similar among the tested five fabrics, which proved that all the samples (with the same surface dimensions 200 × 50 mm^2^) in this study have 25 ends binding warp yarns. It showed that the total crimp angles are the sum of all the repeat unit cells in the fabric. It can be seen that the F2 fabric has the highest crimp angles among the other fabrics. In contrast, the F5 has the lowest crimp angle. The rank of the total crimp angles (F2 > F4> F1 > F3 > F5) is very similar to the friction load discussed in Figure 8. The friction effects on the interface between binding warp and weft yarns depend on the normal force and also the contact surface related directly to the total crimp angle. 

## 4. Conclusions

To quantify the influence of binding path upon the strength transfer from tow to a 3D warp interlock reinforcement and finally to a composite, five different 3DWIFs were conceived and manufactured with the same warp and weft densities. As one of the most important mechanical properties, the tensile properties of these 3DWIFs were experimentally characterised in both warp and weft directions. The inter-yarn frictions in the tensile tests were analysed. Based on Peirce’s geometrical model, the crimp angles between binding warp and weft yarns in 3DWIF structures were calculated and the influence of these angles on the inter-yarn friction was discussed. It can be noted that the warp binding path influences not only mechanical properties in the warp direction, but also the weft direction. The arrangement of weft tows, i.e., their reorganisation and resultant fibreless voids depend on the warp binding path. The binding depth of interlocking warp plays an important role in determining the efficiency of the reinforcement in the loading direction. Increasing binding depth, i.e., crimp amplitude of interlocking warp tows reduces the breaking strength of the fabric in the warp direction. Moreover, the crimp angles/total crimp angles of the 3DWIFs depend on the woven patterns, which are related to the inter yarn friction of 3D fabrics and also influence the tensile properties of 3DWIFs. To predict the tensile deformation and the 3DWIF forming behaviour, the numerical simulation analysis will be one of the future works for the optimisation of the 3DWIF’s architecture.

## Figures and Tables

**Table 1:** Main physical properties of high-molecular-weight polyethylene (HMWPE) yarn.

Product Family	Linear Density (Tex)	Density (g/cm^3^)	Elongation (%)	Breaking Strength (N)
Spectra^®^ 900	135	0.97	4.12 ± 0.01	350.69 ± 6.13

**Table 2:** The structural parameters of five 3-dimensional warp interlock fabrics (3DWIFs) tested.

Fabrics	F1	F2	F3	F4	F5
Architecture	A-L 3-2 4 Binding {Twill 4 effect left}-Stuffer	O-L 3-2 4 Binding {Twill 4 effect left}-Stuffer	A-L 5-3 4 Binding {Twill 6 effect left}-Stuffer	O-T 5-4 4 Binding {Twill 6 effect left}-Stuffer	A-T 5-4 4 Binding {Twill 6 effect left}-Stuffer
Cross-section weft yarns view	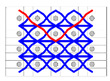	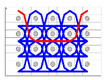	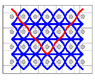	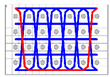	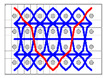
3D view	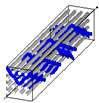	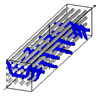	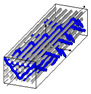	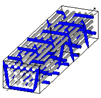	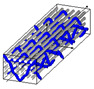
Binding step (X)	3	3	5	5	5
Binding depth (Y)	2	2	3	4	4

**Table 3:** The manufacturer’s specifications of all the dry fabrics.

Fabrics	F1	F2	F3	F4	F5
Warp density (ends/cm)	10				
Weft density (picks/cm)	42				
Thickness (mm)	2.5 ± 0.3	2.1 ± 0.1	1.9 ± 0.1	1.7 ± 0.1	1.6 ± 0.1
Areal weight (g/m^2^)	735.8 ± 36.5	720.0 ± 12.3	688.7 ± 13.2	710.6 ± 12.2	714.1 ± 9.6
FVF (%)	30.3 ± 1.5	35.3 ± 0.6	37.4 ± 0.7	40.7 ± 0.7	46.0 ± 0.6

**Table 4:** Average crimp values of all warp and weft yarns for all the dry 3D warp interlock fabrics.

N° of Fabrics		F1	F2	F3	F4	F5
Warp yarns crimps/(%)	Binding warp yarns crimps%					
	Inter-ply 0	0.85 ± 0.78	1.27 ± 0.27	1.04 ± 0.53	5.78% ± 0.57	4.4 ± 1.06
	Inter-ply 1	1.20 ± 0.44	1.54 ± 0.54	0.93 ± 0.57	-	-
	Inter-ply 2	1.20 ± 0.62	1.49 ± 0.45	-	-	-
	Average value	1.08 ± 0.61	1.43 ± 0.42	0.99 ± 0.55	5.78% ± 0.57	4.4 ± 1.06
	Stuffer warp yarns crimps%					
	Inter-ply 1	0.68 ± 0.28	1.23 ± 0.47	1.28 ± 0.66	4.30 ± 0.58	0.8 ± 0.54
	Inter-ply 2	0.72 ± 0.52	1.20 ± 0.51	0.80 ± 0.50	4.00 ± 0.02	1.33 ± 0.48
	Inter-ply 3	0.93 ± 0.74	1.13 ± 0.39	0.88 ± 0.44	4.18 ± 0.35	0.73 ± 0.30
	Average value	0.78 ± 0.51	1.19 ± 0.46	0.99 ± 0.53	4.16 ± 0.32	0.95 ± 0.44
Weft yarns crimps/(%)	Inter-ply 1	1.24 ± 0.27	0.78 ± 0.49	1.23 ± 0.41	1.97 ± 0.91	1.91 ± 0.36
	Inter-ply 2	1.64 ± 0.30	0.87 ± 0.45	1.42 ± 0.46	1.76 ± 0.52	2.14 ± 0.71
	Inter-ply 3	1.80 ± 0.60	0.72 ± 0.34	1.09 ± 0.40	1.78 ± 0.56	2.00 ± 0.69
	Inter-ply 4	1.38 ± 0.60	0.62 ± 0.37	1.38 ± 0.59	2.03 ± 0.83	1.73 ± 0.40
	Average value	1.52 ± 0.44	0.75 ± 0.41	1.28 ± 0.47	1.89 ± 0.71	1.95 ± 0.54

**Table 5:** Ultimate force (S_t_) and failure value (ε) of fabrics in the warp direction and weft direction.

	Parameters	F1	F2	F3	F4	F5
Warp	S_t_ (kN)	11.2 ± 1.1	14.2 ± 1.0	11.9 ± 0.4	11.9 ± 0.2	6.0 ± 0.5
	ε (%)	4.97 ± 0.01	5.22 ± 0.01	5.39 ± 0.01	10.15 ± 0.01	5.73 ± 0.01
Weft	S_t_ (kN)	48.46 ± 0.1	42.7 ± 1.8	50.3 ± 1.8	51.7 ± 0.5	48.7 ± 3.7
	ε (%)	6.73 ± 0.01	7.10 ± 0.01	6.68 ± 0.01	8.70 ± 0.01	8.37 ± 0.01

**Table 6:** The crimp angles between binding warp and weft yarns and the yarn interlacing shapes in different fabrics.

Fabrics	F1		F2		F3		F4		F5	
Weft cross-Section	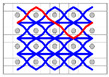		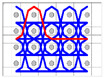		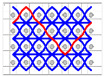		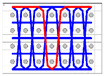		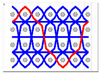	
Single binding warp yarn	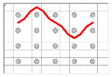		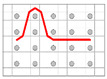		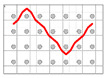		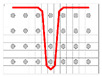		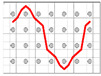	
Cross-linking shape and crimp angles (°)	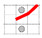	0	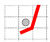	80	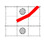	0	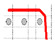	90	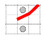	0
	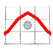	90	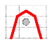	160	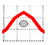	90	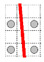	0	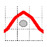	80
	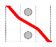	0	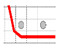	80	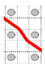	0	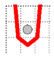	180	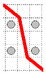	0
	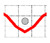	90			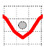	90	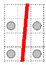	0	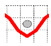	80
							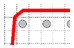	90	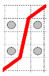	0
Total crimp angles per unit cell (°)	180		320		180		360		160	
Total crimp angles in fabric (×360°)	656.3		1166.7		437.5		875		388.9	
